# Highly Sensitive Force Sensor Based on High-Q Asymmetric V-Shaped CaF_2_ Resonator

**DOI:** 10.3390/mi15060751

**Published:** 2024-06-02

**Authors:** Deyong Wang, Jiamin Rong, Jianglong Li, Hongbo Yue, Wenyao Liu, Enbo Xing, Jun Tang, Jun Liu

**Affiliations:** 1 Key Laboratory of Dynamic Testing Technology, School of Instrument and Electronics, North University of China, Taiyuan 030051, Chinaliuj@nuc.edu.cn (J.L.); 2 School of Semiconductors and Physics, North University of China, Taiyuan 030051, China

**Keywords:** WGM resonator, force sensor, asymmetric V-shaped

## Abstract

Whispering gallery mode (WGM) resonators have high-quality factors and can be used in high-sensitivity sensors due to the narrow line width that allows for the detection of small external changes. In this paper, a force-sensing system based on a high-Q asymmetric V-shaped CaF_2_ resonator is proposed. Based on the dispersion coupling mechanism, the deformation of the resonator is achieved by loading force, and the resonant frequency is changed to determine the measurement. By adjusting the structural parameters of the asymmetric V-shaped resonator, the deformation of the resonator under force loading is improved. The experimental results show that the sensitivity of the V-shaped tip is 18.84 V/N, which determines the force-sensing resolution of 8.49 μN. This work provides a solution for force-sensing measurements based on a WGM resonator.

## 1. Introduction

Whispering gallery mode (WGM) resonators have the advantages of high-quality factors, small mode volume, and high energy density [1,2], and they have become an important component in the optical field. With the continuous development of micro–nano processing technology, the WGM mode can be achieved in the resonant cavities of different materials and different structures. For example, semiconductor materials represented by silicon and silicon nitride [3,4] are widely used in optical communication because of their low transmission loss. Polymer materials represented by polymethylmethacrylate (PMMA) [5,6] and polydimethylsiloxane (PDMS) [7,8], which are highly malleable and at the same time have a small Young’s modulus, can be used for cavity quantum electrodynamics research [9,10] and the production of microcavity lasers. [11] The crystal materials represented by calcium fluoride [12,13] and magnesium fluoride [14,15] have controllable processing shapes, which can achieve precise molding under different structures. In addition, the Q value of fluoride crystal resonators [16,17,18] can reach up to 1011 in preparation, so the line width is narrow and can detect small external changes, which can be used for various types of high-sensitivity sensors [19,20,21].

In recent years, force sensors have been widely used in automotive, aerospace industry, bioengineering, health-care, and other fields, leading to in-depth research. Force sensors based on a WGM resonator has the advantages of strong anti-interference ability, fast transmission rate, and high sensitivity, so it has been deeply studied. Several types of force microsensors based on WGM resonators have been reported. For example, A.L. Huston et al. [22] first used WGM resonance to measure mechanical strain. In the study, a cylindrical resonator made of single-mode quartz fiber was used, with a Q value of 10^5^. A WGM shift of ∼0.8 nm was observed at a strain of ∼58. Tindaro Ioppolo et al. [23] reported a micro-optical force sensor based on a WGM resonator using PMMA fabricated into a 960 μm solid hollow sphere, achieving a Q of 10^6^ with a detection sensitivity of 7.644 nm/N. K. Soler-Carracedo et al. [24] reported on WGM force sensors made of oxyfluoride glass microspheres, pointing out the importance of the material’s thermal expansion and thermo-optical coefficient in sensor sensitivity. Y.E. Geints et al. [25] reported a proof-of-concept for a miniature force sensor based on dielectric microspheres coupled to optical field excitation, which has the important advantage of miniaturizing the design, with the force-sensitive element not being in mechanical contact with the WGM resonator.

In previous work, the materials used to fabricate WGM force resonators have mainly focused on semiconductors, polymers, molten amorphous glass, etc. Although it is easy to form the resonator using these materials, the quality factor obtained is small, which can be one of the limiting factors for the sensitivity of the sensor. Therefore, compared with semiconductor materials, polymer materials, and amorphous glass materials, we have selected fluoride crystal materials that allow for the highest possible Q values. A variety of structural types of WGM microcavities have been thoroughly investigated; common structural microcavities include microdisk cavities, microball cavities, micro-ring cavities, microcylindrical cavities, micro-ring core cavities [26,27,28,29,30], etc. After selecting calcium fluoride crystals as the resonator material, we make the resonator structurally asymmetric and V-shaped to explore the effect of the asymmetric V-shaped structures on the sensitivity of the sensors.

In this study, we used the crystalline material CaF_2_ treated using the single-point diamond polishing technique to fabricate resonators with sizes of 4 mm × 0.3 mm. A CaF_2_ resonator force-sensing system based on an asymmetric V-shaped structure is proposed to improve the efficiency of force sensing. The asymmetric V-shaped structure was modeled, and the theoretical results showed that the sensing efficiency of the CaF_2_ resonant force-sensing system is mainly affected by the variation of the resonator radius. The effect of those three parameters, which determine the geometry of V-shaped CaF_2_ resonators, has been simulated, in particular, the impact of different force levels on the resonator radius. The optimal asymmetric V-shaped structure is obtained by simultaneously optimizing the three parameters. Based on the existing experimental conditions and the CaF_2_ resonator fabrication technology, an asymmetric V-shaped CaF_2_ resonator was successfully fabricated, and the sensitivity at the tip of the asymmetric V-shaped structure is measured to be 18.84 V/N, while the sensitivity at the slightly downward position of the tip is measured to be 9.62 V/N. Therefore, this work provides a support for force sensing based on CaF_2_ V-shaped resonators.

## 2. Fabrication

**Figure 1:**
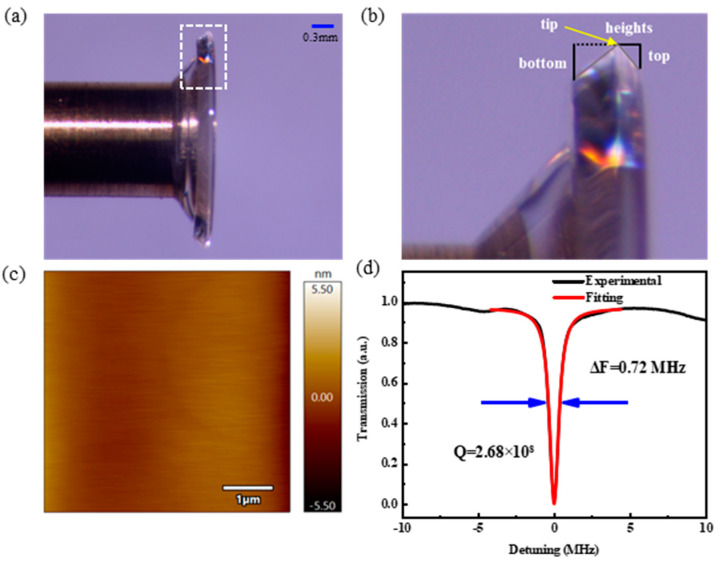
(**a**) Top view of the polished resonator under optical microscope. (**b**) Enlarged view of the area in which the white box is located in (**a**), with the asymmetric V-shaped structure of the area marked. (**c**) Surface morphology of polished resonators under atomic force microscopy. (**d**) Normalized transmission spectra of fabricated asymmetric V-shaped CaF_2_ resonators.

The process of preparing the CaF_2_ resonator is mainly divided into three steps, which are cleaning; gluing the resonator; and single-point diamond turning and polishing. In the first step, the resonator is cleaned with an ultrasonic cleaner to achieve a high degree of cleanliness on the resonator surface to ensure that the bonded resonator is stable. In the second step, a copper post is attached to the fixture and the surface of the copper post is coated evenly with UV glue. The knob on the top of the fixture was turned to move the copper post toward the resonator until it made contact, then it was irradiated with a UV lamp. The bonded resonator is well secured to the air-bearing spindle with a copper post. In the third step, the molding of the crystal resonator is completed by single-point diamond turning. However, some defects may exist on the surface of the crystal. By successively reducing the size of the polishing particles and smoothing the surface by fine polishing, the roughness of the crystal surface is improved. Through the above three processing steps, resonators with low surface roughness, transparent edges, and no scratch damage can be obtained, as shown in Figure 1a. We zoomed in on the region where the white box is located in Figure 1a and labeled the asymmetric V-shaped structure in this region. The yellow arrow points to the position of the “tip”, where the black solid lines represent the structure contour parameters “top”, “heights”, and “bottom”, as shown in Figure 1b. The surface topography was measured using atomic force microscopy (AFM) and the surface roughness, Ra = 0.484 nm, was calculated, as shown in Figure 1c. The surface roughness affects the quality of the resonator, and its optical quality factor is evaluated by measuring the line width of the coupling curve. The line width is 0.72 MHz, and the Q value of the crystal resonator is calculated to be 2.68 × 10^8^. The red line is the Lorentzian fit, and the black line is the actually measured data, as shown in Figure 1d.

## 3. Theoretical Analysis and Discussion

**Figure 2:**
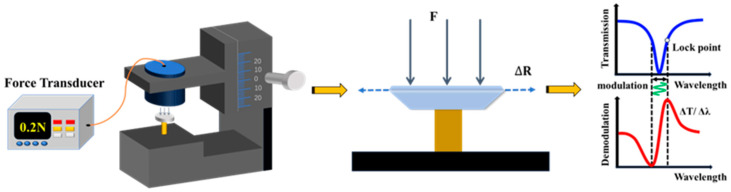
Experimental schematic of force sensing based on asymmetric V-shaped resonator.

We use the finite element method to optimize the sensing efficiency by adjusting the parameters of the resonator structure. According to the simulation results, we finally fabricated the asymmetric V-shaped structure with the contour parameters of top = 50 µm, height = 50 µm, and bottom = 700 µm, and the optimization process is discussed in detail in Section 4. The CaF_2_ resonator we chose has a diameter of 4 mm and a thickness of 0.3 mm, and the asymmetric V-shaped slit was customized to facilitate the re-manufacturing of this structure resonator. A force sensor based on a high-Q asymmetric V-shaped CaF_2_ resonator is schematically shown in Figure 2, where the CaF_2_ resonator can be used for optical input and output through a tapered optical fiber. In the force sensing system, the CaF_2_ resonator is pressurized using a helical microdisplacement pressurization device, and the CaF_2_ resonator is deformed by the external load, which also changes its optical resonance parameters, resulting in a shift in the resonance wavelength. In order to improve the detection accuracy, the force signal is detected by locking the laser frequency at the maximum slope of the resonance side by using the PDH locking technology. The demodulated signal is a differentiation of the transmission spectrum

From the standing wave condition of the WGM, the resonance wavelength can be determined as follows:

(1) $$\lambda_{r}=\frac{2\pi Rn_{eff}}{m}$$

where *m* is the resonant mode order and *λ_r_* is the resonance wavelength. *n_eff_* and *R* are the effective refractive index and radius of the CaF_2_ resonator, respectively. In the force sensing system, the CaF_2_ resonator changes its radius, as well as its effective refractive index, under external force, causing the resonance wavelength to shift, which can be expressed as follows:

(2) $$\frac{\Delta \lambda_{r}}{\lambda_{r}}=\frac{\Delta R}{R}+\frac{\Delta n_{eff}}{n_{eff}}$$

**Figure 3:**
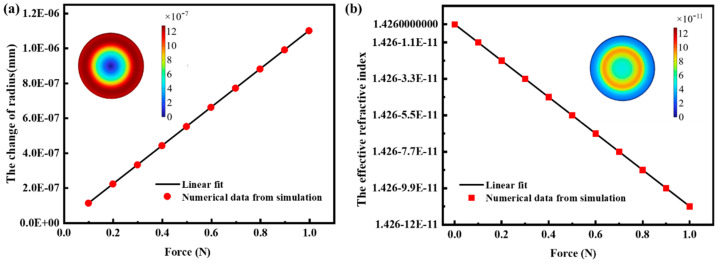
(**a**) Varying *R* for different forces simulated. The inset shows the deformation of *R* when subjected to a force of 1 N. (**b**) The variation of *n_eff_* with applied force under simulation. The inset shows the resonator strain at 1 N force.

The finite element method (FEM) was used to simulate the radius and refractive index variations of asymmetric V-shaped CaF_2_ resonators at different forces. The basic properties of the material are as follows: density is 3.18 g/cm^−3^, Young’s modulus is 75.8 Gpa, and Poisson’s ratio is 0.26. The diameter is 4 mm and the thickness is 0.3 mm. The first influencing factor is the variation of the radius; in the force sensing system, the light is coupled to the CaF_2_ resonator and circulates along the circumference, so we choose the variation of the outermost radius. As shown in Figure 3a, the variation of the CaF_2_ resonator radius at different forces has a good linear relationship with a slope of 1.07 × 10^−6^ mm/N. The inset shows the effect of a force of 1 N on the variation of the CaF_2_ resonator radius. Another influencing factor is the change in refractive index. The change in refractive index due to the stress in the CaF_2_ resonator is given by the following equation:

(3) $$n_{1}=n_{2}={\left(n_{0}^{-2}+p_{12}\right)}^{-\frac{1}{2}}$$

where *n*_0_ = 1.426 is the refractive index of the CaF_2_ resonator under ambient conditions and *p*_12_ = 0.198 is the elastic optical coefficient. Using FEM, the relationship between the refractive index of the CaF_2_ resonator and the force is obtained by applying different forces. The refractive index has a good linear relationship with force, with a slope of 1.1 × 10^−10^/N, as shown in Figure 3b. The inset illustrates the effect of a 1 N force on straining a CaF_2_ resonator. Through the above simulations, the effects of CaF_2_ resonator radius change and refractive index change on the resonance wavelength shift were obtained. The resonance wavelength shift due to the radius change is 8.277 × 10^−4^ nm/N, and the resonance wavelength shift due to the refractive index change is 1.196 × 10^−7^ nm/N. Therefore, it can be concluded that the resonance wavelength shift is mainly affected by the radius change, and the effective refractive index change has a negligible effect on the resonance wavelength shift.

The sensitivity of a force-sensing system based on an asymmetric V-shaped CaF_2_ resonator can be defined as follows:

(4) $$S=\frac{\Delta T}{\Delta F}=\frac{\Delta T}{\Delta \lambda_{r}}=\frac{\Delta \lambda_{r}}{\Delta F}$$

where *T* is the output signal, *F* is the force, and *λ_r_* is the resonance wavelength. The first term Δ*T*/Δ*λ_r_* is the slope of the resonance spectrum. It is closely related to the Q of the WGM resonator; the larger the Q, the larger the slope and the higher the sensitivity. The second term Δ*λ_r_*/Δ*F* is the force sensing efficiency of the WGM resonator, i.e., the resonance wavelength shift caused by the change in *R* and the change in *n_eff_* of the CaF_2_ resonator. Since the resonance wavelength shift caused by the change of *n_eff_* is negligible, the force sensing efficiency can be expressed as follows:

(5) $$\Delta \lambda_{r}/\Delta F=\lambda_{r}(\Delta R/R)$$

## 4. Experimental Setup and Results

**Figure 4:**
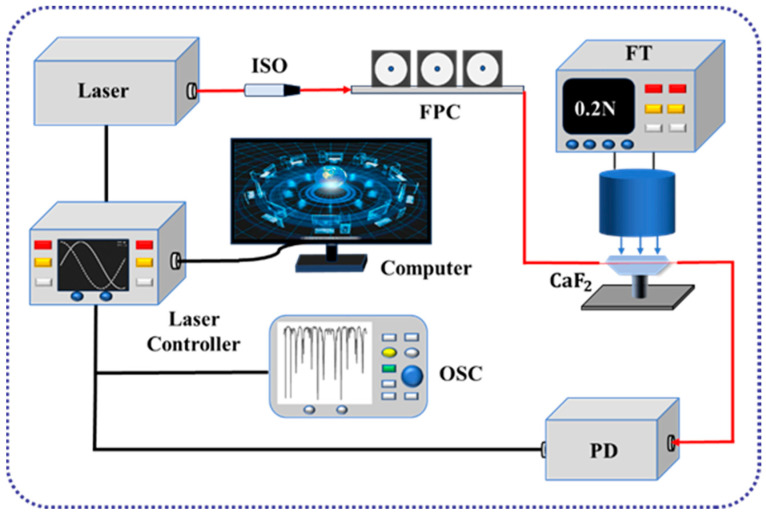
Schematic diagram of the experimental setup for force sensing system. Isolator, ISO; fiber polarization controller, FPC; photodetector, PD; oscilloscope, OSC; force transducer, FT. Lines in red indicate optical connections; lines in black indicate circuit connections.

The experimental setup for the force sensing system is shown in Figure 4. A tunable laser with a linewidth of 10 kHz was used and was tuned to the 1550 nm band. Light is generated by the tunable laser and output through the isolator. The output light passing through the isolator is not reflected back to the laser, which has a protective effect on the laser. The output light is coupled to the CaF_2_ resonator via a tapered fiber, and the coupling strength is optimized using a polarization controller (PC). The coupled output light is connected to a photodetector (PD) through an intensity-adjustable attenuator, and the electrical signal output from the photodetector is transmitted to an oscilloscope (OSC) for signal display and response.

To generate the force signal sensed by the force-sensing system, the sensitive unit of a standard commercial force transducer (FT) is clamped to a three-dimensional translation stage to form a pressurized device. The CaF_2_ resonator is pressurized or depressurized by turning the knob. When the pressurizer knob is turned clockwise, an external load is applied to the CaF_2_ resonator, i.e., the pressurization process; when the pressurizer knob is turned counterclockwise, the force is released from the CaF_2_ resonator, i.e., the structure tends to a zero force state. This pressurized device generates force, while simultaneously measuring the amount of force applied by a standard commercial force transducer. Force deforms the resonator, changing the optical parameters and providing a smooth reference line on the oscilloscope through the laser’s own frequency-locked module. The effect of the force signal on the optical resonant frequency is converted into a change in response amplitude for more intuitive and reliable force detection.

**Figure 5:**
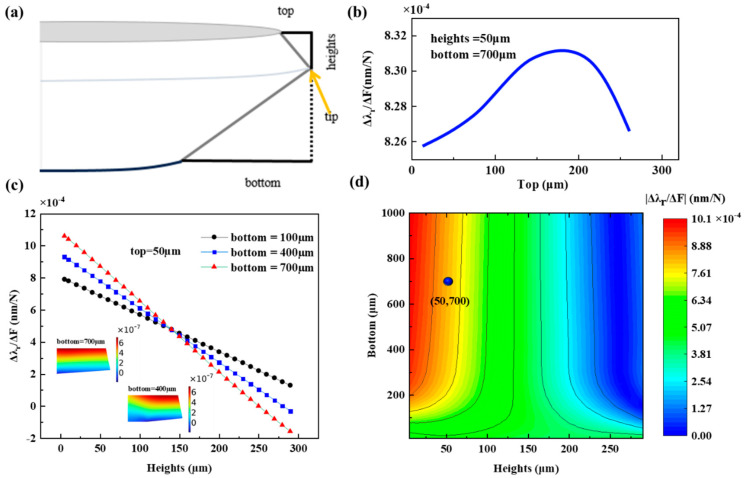
(**a**) Asymmetric V-shaped profile of CaF_2_ resonator. (**b**) The Δ*λ_r_*/Δ*F* of the CaF_2_ resonator as a function of ‘top’ under simulation. (**c**) The Δ*λ_r_*/Δ*F* of the CaF_2_ resonator as a function of ‘heights’ under simulation. Inset: the ∆*R*/*R* distributions of the resonator at bottom = 400 (700) µm. (**d**) The |Δ*λ_r_*/Δ*F*| of the resonator as a function of ‘heights’ and ‘bottom’ for top = 50 μm under simulation. Blue sphere: structural parameters of the fabricated resonator.

In order to improve the sensitivity of the force sensing system, on the one hand, we used a single-point diamond turning method to fabricate a high-Q CaF_2_ resonator; on the other hand, we need to improve the efficiency of the force sensing. For this purpose, we designed an asymmetric V-shaped structure CaF_2_ resonator. The CaF_2_ resonator has a diameter of 4 mm and a thickness of 0.3 mm, and its asymmetric V-shaped profile is determined by the following three parameters: top, height, and bottom, as shown in Figure 5a. Theoretically, the structural parameters of the asymmetric V-shaped resonator can modulate the deformation of the resonator under force. We used the finite element method to analyze the effects of the three parameters of the asymmetric V-shaped structure on Δ*R*/*R*, and then derived how the three parameters affect the efficiency of force sensing using Equation (5).

Firstly, the V-shaped structure parameter ‘top’ of the resonator was analyzed. A total of 1 N force was applied to the force sensing system, heights = 50 µm, bottom = 700 µm, and the influence of the parameter ‘top’ on the sensing efficiency (Δ*λ_r_*/Δ*F*) at different values was simulated, as shown in Figure 5b. The influence of the value of ‘top’ on Δ*λ_r_*/Δ*F* is less than 0.7%, and the main role of ‘top’ in the V-shaped structure is to constrain the distribution of optical modes. Based on the above model parameters, the effect of heights on the Δ*λ_r_*/Δ*F* of the resonator is observed by partially taking the value of ‘bottom’, while keeping top = 50 µm. The trends of Δ*λ_r_*/Δ*F* for different values of heights are shown in Figure 5c for bottom = 100 µm, 200 µm, 400 µm, and 700 µm, respectively. From the figure, it can be seen that Δ*λ_r_*/Δ*F* decreases with increasing heights and changes faster with increasing bottom. In addition, we can see that the value of Δ*λ_r_*/Δ*F* appears to be less than 0 for the bottom = 400 (700) µm. The insets in Figure 5c show the Δ*R*/*R* distribution of the resonator at bottom = 400 (700) µm. It can be seen that the strain in the blue part is less than 0, which means that the resonator shrinks in the radial direction under external force. The part outside the blue color has a strain greater than 0, indicating that the resonator expands outward under external force. The direction of deformation of the resonator determines whether Δλ_r_/ΔF is positive or negative.

From the above two simulation results, it can be seen that in the asymmetric V-shaped structure of the CaF_2_ resonator, the effect of top on Δ*λ_r_*/Δ*F* is extremely small; heights and bottom have good linear relationships with Δ*λ_r_*/Δ*F*, which clearly demonstrates the influence of the three parameters on the performance of the CaF_2_ resonator. In order to find the optimal combination of parameters to improve the efficiency of force sensing, we fix the parameter top = 50 µm and simulated the values of the other two parameters to obtain the function of |Δ*λ_r_*/Δ*F*| on heights and bottom. The simulation results show that |Δ*λ_r_*/Δ*F*| is larger when heights is smaller and the bottom is larger, as shown in Figure 5d.

**Figure 6:**
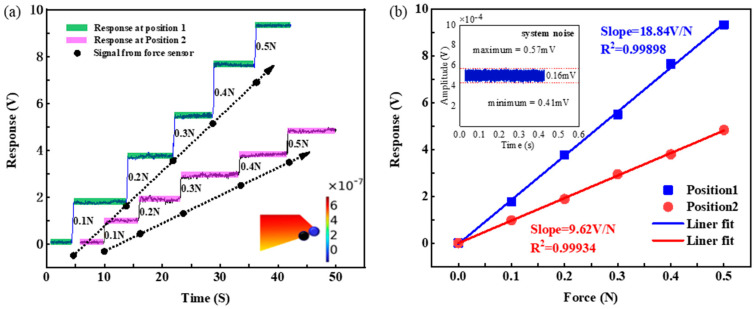
(**a**) Voltage output response of CaF_2_ resonator under forces at two different positions. Inset: the coupling positions and the distribution of Δ*R*/*R*. (**b**) The voltage output response depends on the applied force in the case shown in (**a**). Inset: system noise.

Considering the distribution of the optical mode field and the limitations of the resonator preparation process, we finally made the profile parameters of the resonator V-shaped structure as top = 50 µm, heights = 50 µm, and bottom = 700 µm, respectively, as shown by the blue sphere in Figure 5d. The force-sensing efficiency at the tip of the V-shaped structure of this resonator is simulated to be Δ*λ_r_*/Δ*F* = 8.28 × 10^−4^ nm/N. An experimental validation was performed to test an asymmetric V-shaped CaF_2_ resonator as a force-sensing element, in which a pressurized device was used to apply force to the resonator. We experimentally measured at two positions under this structure, recording several values of applied force and the corresponding voltage responses. As shown in Figure 6a, the resonator was continuously pressurized with the applied force ranging from 0.1 N to 0.5 N, and the experimental results of applying incremental force in 0.1 N increments were tested; it was observed that the WGM resonator variations exhibited a voltage output response that showed a step change trend with increasing force, and the voltage output response time is short in the test load range, thus providing reliable force readings that are important to the performance of the sensor. Position 1 is at the tip of the asymmetric V-shaped structure and position 2 is slightly downward of the tip, as shown in the inset of Figure 6a. We measured the voltage output response at two positions under different forces. The two positions have almost the same Q factor, but the response at position 1 is significantly stronger than that at position 2. The corresponding voltage output response as a function of applied force is plotted as shown in Figure 6b, which is obtained by linear fitting with a slope of 18.84 V/N and a goodness of fit r^2^ = 0.99898 at position 1, and a slope of 9.62 V/N and an r^2^ = 0.99934 at position 2. The sensitivity at position 1 is 1.96 times that at position 2. It shows that the design of the asymmetric V-shaped structure can improve the sensitivity of force sensing. It is important to note that for all force measurements discussed here, the temperature of the environment surrounding the sensing system is kept constant to avoid additional effects of temperature variations on the output response. In addition, sensor sensitivity is not the only measure of the device’s detection capability; to fully characterize the sensor’s performance, we also need to know the sensor’s detection limit. For the force sensing we are investigating, the detection limit represents the smallest change in external force that can be detected. As shown in the inset of Figure 6b, the noise of the system without an applied external force is demonstrated with a noise amplitude of 0.16 mV. By calculation, the minimum detectable force is 8.49 µN with a system sensitivity of 18.84 V/N.

## 5. Conclusions

This paper investigates the sensing mechanism of a CaF_2_ resonator based on the asymmetric V-shaped structure of the resonator, and analyzes the effects of varying the resonator radius and effective refractive index on the resonance wavelength shift of the force sensing system. To improve the force sensing efficiency of the sensor, the effects of the three parameters top, heights, and bottom, which determine the asymmetric V-shaped profile, on |Δ*λ_r_*/Δ*F*| under external force are simulated and analyzed. Trends in force-sensing efficiency were obtained and a physical explanation for these trends was provided. Through simulation analysis, the asymmetric V-shaped structure was optimized to enhance the coupling strength between the force and light fields, and the best parameter combination to improve the force sensing efficiency was obtained. The force sensing sensitivity of the resonator is measured to be 18.84 V/N at the tip of the asymmetric V-shaped structure and 9.62 V/N at the slightly downward tip, based on the existing experimental conditions and fabrication technique of the CaF_2_ resonator. It is proven that the V-shaped structure plays a crucial role in sensitivity enhancement. In addition, we tested the system with a minimum detectable external force of 8.49 µN. It provides a new solution for force measurement using the WGM CaF_2_ resonator as a platform for a wide range of applications.

## Data Availability

Data underlying the results presented in this paper are not publicly available at this time but may be obtained from the authors upon reasonable request.
